# Occurrence of Microcystins and Toxin-Producing Cyanobacteria in Reservoirs of the Alqueva Water Storage System (Southern Portugal)

**DOI:** 10.3390/toxins18100430

**Published:** 2026-10-07

**Authors:** Tomé Azevedo, Lucas Ribeiro, Joana Azevedo, Rui Barbosa, Rita Mendes, Joana Martins, Marisa Freitas, Vitor Vasconcelos, Alexandre Campos

**Affiliations:** 1 CIIMAR/CIMAR LA, Interdisciplinary Centre of Marine and Environmental Research, University of Porto, Terminal de Cruzeiros do Porto de Leixões, 4450-208 Matosinhos, Portugal; atomail@hotmail.com (T.A.); lil.ribeiro96@gmail.com (L.R.); joana.azevedo@ciimar.up.pt (J.A.); rui.fmbarbosa23@gmail.com (R.B.); rmendes@ciimar.up.pt (R.M.); maf@ess.ipp.pt (M.F.); vmvascon@fc.up.pt (V.V.); 2 Industrial Design Engineering, Delft University of Technology, Landbergstratt 15, 2628 CE Delft, The Netherlands; j.martins@tudelft.nl; 3 School of Health, Technical University of Porto, Rua Dr. António Bernardino de Almeida, 400, 4200-072 Porto, Portugal; 4 Biology Department, Faculty of Sciences, University of Porto, Rua do Campo Alegre, 4169-007 Porto, Portugal

**Keywords:** Alqueva reservoirs, cyanobacterial blooms, cyanotoxins, microcystins, water quality

## Abstract

Cyanobacterial blooms and the associated production of cyanotoxins represent a growing concern for freshwater ecosystems, particularly in regions increasingly affected by eutrophication and climate change. In Portugal, data on the occurrence and distribution of cyanotoxins remain limited, especially in southern multipurpose reservoirs that support the water supply and irrigation in a region severely affected by water scarcity. This study investigates the presence of cyanobacteria and their major cyanotoxin groups—microcystins (MCs), cylindrospermopsin (CYN), and anatoxin-a (ANTX)—in three reservoirs of the Alqueva water storage system (EFEMA), combining chemical analyses with molecular tools to assess toxin-producing cyanobacteria. Liquid chromatography–mass spectrometry (LC–MS) detected MC-LR, MC-RR, and MC-YR exclusively in the particulate fraction of water samples, while no toxins were detected in the dissolved fraction. MC concentrations were generally low (<0.1 µg/L), with the highest levels observed during summer, particularly in July. Among the variants, MC-LR was the most common and abundant. Molecular analyses (16S rRNA) confirmed the widespread presence of cyanobacteria and identified the genus *Microcystis* in all reservoirs. Several genes from the microcystin biosynthesis cluster (*mcyA*, *mcyB*, *mcyD*, *mcyE*, and *mcyG*) were amplified, and sequencing of the *mcyA* gene revealed high homology with *Microcystis aeruginosa*. Although the detected MC concentrations remained below World Health Organization (WHO) guideline values for drinking water, their recurrent detection and the confirmed presence of toxin-producing genotypes highlight the need for continued monitoring. This study underscores the value of integrating chemical and molecular approaches for assessing cyanotoxin risks in large multipurpose reservoir systems under increasing environmental pressure.

## 1. Introduction

Cyanobacteria constitute a highly diverse group of prokaryotic microorganisms that inhabit a large variety of ecosystems and comprise an important component of phytoplankton. Unlike other prokaryotes (bacteria and archaea), cyanobacteria perform aerobic photosynthesis using chlorophyll-a as their primary photosynthetic pigment [1]. They play key ecological roles by fixing atmospheric nitrogen, producing oxygen, and serving as primary producers in aquatic food webs. Eutrophication, driven by anthropogenic activities, is creating favorable conditions for the proliferation of these microorganisms in many aquatic environments. This cyanobacteria proliferation is also exacerbated, nowadays, by climate change. In addition, some cyanobacterial species are considered harmful due to the production of toxic compounds (cyanotoxins). These toxic cyanobacteria, when present at high cell densities, can severely impair water quality, posing a threat to ecosystems, animals, and humans [2].

The primary routes of human exposure to cyanotoxins include the ingestion of contaminated water or food and dermal exposure. Microcystins (MCs) are the most diverse and frequently reported cyanotoxins in freshwater environments and are recognized for their acute toxicity to animals and humans [2]. MCs are a group of highly variable cyclic peptides with a common structure comprising cyclo-(D-Ala^1^-X^2^-D-Masp^3^-Z^4^-Adda^5^-D-γ-Glu^6^-Mdha^7^), in which X and Z correspond to variable amino acids [3]. Both acute and chronic exposures to MCs have been associated with adverse health effects, particularly liver injury mediated through the inhibition of protein phosphatases [4]. Other cyanotoxins frequently detected in water bodies include cylindrospermopsin (CYN), anatoxin-a (ANTX), and saxitoxins (STX). ANTX and STX affect primarily the nervous system and are therefore classified as neurotoxins, whereas CYN can damage multiple organs and is therefore considered cytotoxic [1,5].

To protect public health, guideline values for MC concentrations in drinking and recreational waters have been established. The World Health Organization recommends limits of 1 µg MC-LR /L for chronic (lifetime) exposure in drinking water, 12 µg MC-LR /L for short-term drinking water exposure, and 24 µg MC-LR /L for recreational waters. As for other cyanotoxins (ANTX, CYN, STX), provisional short-term drinking water health-based reference values and provisional recreational water health-based reference values were derived. Given the lack of scientific supporting data, these values should be used with caution when establishing regulations or standards [6].

The first identification and characterization of MCs in Portuguese freshwater ecosystems occurred in the 1990s. *Microcystis aeruginosa* was identified as the species responsible for MC production in northern Portugal [7]. The detected MC variants included MC-LR, MC-RR, MC-YR, and [D-Asp^3^]MC-LR [8]. Pereira et al. (2000) reported the presence of STX associated with *Aphanizomenon flos-aquae* in the Montargil reservoir (central Portugal) [9]. Later, Saker et al. (2004) [10] documented the occurrence of toxic *Cylindrospermopsis raciborskii* strains in Portuguese freshwaters. CYN, and the toxic genus *Aphanizomenon* were reported in Central Portugal in 2012–2013 [11]. Historical reports of potential toxic cyanobacteria in Portugal and other Mediterranean countries (Spain and Italy) were recently reviewed by de Figueiredo et al. (2025) [12] and Pinto et al. (2026) [13]. These studies demonstrate that cyanobacteria are widely spread in freshwater sources in Southern European countries with cell densities frequently exceeding the WHO medium-risk drinking level (20,000 cells/mL).

Reports of cyanotoxins in Portuguese freshwater systems remain scarce. Moreira et al. (2020) documented MC concentrations between 1.6 and 18.8 μg/L in several freshwater bodies in northern and central Portugal [14]. Less frequently detected were the ANTX and STX [14]. Another study by Menezes et al. (2017) in recreational waters in central Portugal found persistent MCs contamination [15]. Although concentrations generally did not exceed 4 μg/L, occasionally, high concentrations above 20 μg/L were observed. Considering the country’s vulnerability to cyanobacterial proliferation, further studies are needed concerning the geographical distribution, diversity, dynamics, and evolution of toxic cyanobacteria and cyanotoxins. Such information is essential to improve risk assessment and current freshwater resources management practices.

The Alqueva water storage system, located in the Alentejo region of Southern Portugal, was built to supply freshwater to populations in an area frequently affected by severe water scarcity. It is the largest strategic water reserve in Europe and supports a major portion of agricultural activity in the region. The system comprises 72 reservoirs (including the main Alqueva reservoir), interconnected by a complex network of waterways and canals that supply water to approximately 10,000 km^2^ across 20 municipalities in the districts of Beja, Évora, Portalegre, and Setúbal [16]. Water quality in Alqueva has been negatively affected by prolonged droughts, high temperatures, and intensive agriculture. Despite concerns about the occurrence of toxic cyanobacteria, monitoring studies remain limited. Rodrigues et al. (2013) [17] reported total MC concentrations in the main reservoir, with the highest values occurring during the warmest months (June, July, and September). MC-LR and MC-RR concentrations ranged from 17 to 344 ng/L and 25–212 ng/L, respectively.

The aim of the present work was to assess, through chemical methods, the occurrence of the main groups of cyanotoxins (MCs, CYN, ANTX) in three reservoirs located in the Beja region (São Pedro, Magra, and Pisão) and to evaluate water quality based on these parameters. Additionally, the presence of toxin-producing cyanobacteria was investigated using molecular biology tools.

## 2. Results

### 2.1. Cyanotoxins Screening

We first performed a full LC-MS scan to screen for the presence of several cyanotoxins in the three sampled reservoirs. The analysis comprised three MC variants (MC-LR, MC-RR, and MC-YR), as well as CYN and ANTX. MCs were detected only in the particulate fractions of the samples. No toxins were detected in the dissolved fractions. Furthermore, neither CYN nor ANTX were detected in any of the analyzed samples by LC–MS (Table 1). All three MC variants (MC-RR, MC-LR, and MC-YR) were detected in the three reservoirs (São Pedro, Magra and Pisão), although they did not always co-occur during the same month (Table 1). Among the detected variants, MC-LR appeared to be the most persistent across the investigated reservoirs.

### 2.2. Microcystins Quantification

Following toxin screening, a targeted LC–MS method was applied to quantify the individual toxin variants. MC concentrations were consistently below 0.1 µg/L, except in September 2020 in São Pedro reservoir (Table 2). Among the three reservoirs, São Pedro showed the highest MC-LR concentration (0.115 ± 0.131 µg/L), while Pisão exhibited the lowest (0.031 ± 0.002 µg/L) (Table 2). MC-LR was the most frequently detected MC variant at the highest concentrations. MC-YR was quantified in only four samples, originating from the Pisão and Magra reservoirs. July was consistently the month with the highest MC-LR concentrations across all reservoirs, except in August 2020, when the highest MC-LR concentration was recorded in the Magra reservoir (Table 2). The details of the LC–MS analysis for the quantification of MCs are reported in the Supplementary Material (Figure S1).

### 2.3. Molecular Identification of Microcystis

The detection of cyanobacterial DNA in the water samples from 2020, was carried out by PCR and amplification of 16S rRNA gene, with 27F/809R and 740F/1494R [18] primer pairs, and the phycocyanin operon with PCβF/PCβR primer pair [19]. For the detection of *Microcystis* specific genes, primers Micr 184F/Micr 431R [18] were applied. Results were positive for both the 16S rRNA gene and the phycocyanin operon (Supplementary Table S3). Furthermore, the amplification of gene sequences specific to the genus *Microcystis*, with the pair of primers Micr 184F/Micr 431R provided further evidence of the presence of this genus in all reservoirs (Table 3). At all sampling points where cyanobacteria were reported, *Microcystis* was also detected using this molecular approach.

Additionally, an analysis of the genes belonging to the microcystin biosynthesis gene cluster (*mcyABCDEG*) was performed. No gene amplification was obtained with the pair of primers used to amplify the gene *mcyC* (PSCF1/PSCR1) [20]. Nevertheless, the primer pairs 2156F/3111R [21], PKDF2/PKDR2, PKDE1/PKER1 [20], and PKGF1/PKGR1 [20] led to the amplification of sequences corresponding to the genes *mcyA*, *mcyB*, *mcyD*, *mcyE*, and *mcyG*, respectively in several of the samples collected in 2020. The results of this analysis are summarized in Table 4.

Sequencing of the *mcyA* gene was attempted in order to identify the taxonomic group present in the environmental samples. From this analysis, it was only possible to obtain high quality sequencing data for the samples collected in July and August 2020 in the S. Pedro reservoir. After a homology search using the BLAST bioinformatics tool https://blast.ncbi.nlm.nih.gov/Blast.cgi (accessed on 23 October 2024), a match was found with the *mcyA* gene sequence from *M. aeruginosa* CP086723.1 (Table 5).

## 3. Discussion

The present study provides new insights into the occurrence of cyanotoxins and toxin-producing cyanobacteria in three reservoirs from the Alqueva water storage system (São Pedro, Magra, and Pisão), during the summer months in the years 2020 and 2021, contributing to the still limited data available for this water system. By combining chemical analyses (LC–MS) with molecular tools (PCR and gene sequencing), this work provides an assessment of both cyanotoxin presence and the underlying biological potential for toxin production.

Microcystins were the only cyanotoxins detected in the particulate fraction of the analyzed samples, whereas CYN and ANTX were not detected in either the particulate or dissolved fractions. This finding is consistent with previous studies in Portuguese freshwater systems, where MCs are the most frequently reported cyanotoxins [7,14], while other toxin classes appear to occur more sporadically and at lower concentrations. The absence of dissolved toxins suggests that, during the sampling period, cyanotoxins were predominantly retained intracellularly, likely reflecting the initial stages of cyanobacterial blooms rather than bloom senescence. This pattern has been commonly observed in systems with moderate cyanobacterial abundance [22].

All three investigated MC variants (MC-LR, MC-RR, and MC-YR) were detected across the reservoirs, although their occurrence varied temporally (Table 2). MC-LR emerged as the most persistent and abundant variant, corroborating its dominance in freshwater ecosystems worldwide and its frequent detection in Portuguese waters [7,14,15]. The sporadic detection of MC-YR, particularly in Pisão and Magra reservoirs, may reflect differences in cyanobacterial community composition, environmental conditions, nutrient concentrations (particularly phosphorous and nitrogen) or strain-specific toxin profiles [23,24]. Seasonal variability was evident, with July generally exhibiting the highest MC concentrations(Table 2), consistent with higher temperatures and enhanced cyanobacterial growth during the summer months, from July to September, as reported by Valério et al. (2008) [22]. The exception observed in August 2020 at the Magra reservoir (Table 2) suggests that local environmental factors, such as nutrient availability or hydrological conditions, may modulate toxin dynamics at smaller spatial scales.

Although MC concentrations were consistently below the WHO guideline value for drinking water (1 µg/L), their repeated detection across reservoirs and sampling periods supports the need for continued monitoring to better characterize their temporal variability. Similar concentration ranges have been reported in other Portuguese reservoirs [15,17]. Even at low concentrations, the long-term presence of MCs may pose ecological risks to aquatic organisms [25,26] and warrants continued monitoring, particularly in multipurpose reservoirs such as Alqueva that support drinking water supply, irrigation, and recreation. In this study, we opted to use a 10% aqueous methanol (MeOH) solution to enable the simultaneous extraction of toxins with different solubility properties. However, this approach may have affected the extraction efficiency of microcystins to some extent, leading to a lower estimation of MC concentrations. Indeed, the recovery of MCs is generally improved when higher MeOH concentrations are used (typically between 40% and 80%) [27]. In this regard, and considering that MCs seem to be the most prevalent toxins in the Alqueva reservoirs, the extraction procedure could be further optimized by increasing the MeOH concentration to improve the recovery and, consequently, the detection of these toxins in future research. The molecular analyses provided complementary evidence supporting the chemical data. The amplification of cyanobacterial 16S rRNA genes and the phycocyanin operon confirmed the presence of cyanobacteria in all reservoirs during 2020. A good agreement was found in the analysis of both gene markers, with both being useful for the detection of cyanobacteria. Although not employed in this case, Phycocyanin operon analysis could be more effective than 16S rRNA in resolving close relationships and interspecies variability [28]. Furthermore, the successful amplification of *Microcystis*-specific gene sequences indicates that this genus is a common component of the cyanobacterial community in the studied systems. This identification is in agreement with the detection of MC-LR in July and August 2020 in the São Pedro reservoir, highlighting its possible role as the primary MC-producing taxon in this system. This finding also aligns with earlier reports identifying *Microcystis aeruginosa* as a major MC producer in Portuguese freshwater environments [7].

The detection of multiple genes from the microcystin biosynthesis cluster (*mcyA*, *mcyB*, *mcyD*, *mcyE*, and *mcyG*) in several samples provides strong evidence for the genetic potential for MC production in these reservoirs. The absence of amplification of the *mcyC* gene may be attributed to primer specificity, sequence variability among environmental strains, or low gene copy numbers [29,30]. Importantly, sequencing and BLAST analysis of the *mcyA* gene from São Pedro reservoir samples revealed high homology with *Microcystis aeruginosa*, further highlighting its possible role as the primary MC-producing taxon in this system. These molecular findings align with the detection of MC-LR in São Pedro samples from July and August 2020 (Table 2). Whether this is the only species contributing to the toxin profile found in this study is difficult to ascertain with the current data.

Hydrological connectivity can shape cyanobacterial distributions and toxin potentials across interconnected reservoirs. This is consistent with the detection of MCs in both São Pedro and Magra reservoirs at same time, suggesting that water supplied from São Pedro may carry cyanobacterial biomass or genetic potential for toxin production into Magra, rather than independent, isolated bloom development in each reservoir [31].

Water residence time is a key driver of cyanobacterial growth and, consequently, cyanotoxin production [32]. In this study, however, the summers of 2020 and 2021 were characterized by extreme drought conditions, which resulted in intensified water abstraction for public water supply and agriculture. This factor should be considered in future studies to better understand the cyanobacteria community dynamics and the levels of cyanotoxins present in the reservoirs. One plausible explanation for the low concentrations of cyanotoxins found in these reservoirs could indeed be related with the low residence time of the water, which reduces the chances of a cyanobacteria bloom developing, despite the favorable water temperatures registered in these periods (between 21 and 25 °C), close to the thermal growth optimum for, for instance, *Microcystis aeruginosa* (28 and 30 °C) [33]. This mechanism provides a plausible explanation for the low MC concentrations detected, even in the presence of toxin-producing genotypes and favorable temperature conditions [34]. We also recommend that future monitoring should continue beyond the July–September period to include early summer and early autumn, as these periods are also of concern. Continued monitoring over several years would help characterize interannual variability, while more frequent sampling during the monitoring period would help capture short-term changes in cyanobacterial blooms and consequently cyanotoxins concentrations, which are important for water management. Building on the combined chemical and molecular approach used here, monitoring should continue to analyze both dissolved and particulate cyanotoxin fractions. This is particularly relevant because MCs were detected exclusively in the particulate fraction in this study. Monitoring both fractions would also help track toxin release following cell lysis. Chemical measurements would complement microscopic identification, while qPCR targeting MC biosynthesis genes could help track changes in genetic potential for toxin production. However, molecular results should be interpreted alongside measured toxin concentrations, as the presence of biosynthesis genes does not necessarily demonstrate active toxin production.

## 4. Conclusions

This study provides a comprehensive assessment of cyanotoxin occurrence and the presence of toxin-producing cyanobacteria in three reservoirs of the Alqueva water storage system in southern Portugal. Microcystins were the only cyanotoxins detected, with MC-LR, MC-RR, and MC-YR occurring at low concentrations and predominantly in the intracellular fraction. The absence of cylindrospermopsin and anatoxin-a suggests that, during the study period, these toxin classes did not pose a significant risk in the investigated reservoirs.

The systematic detection of MCs, particularly during the summer months, consistent with the molecular identification of *Microcystis* spp. and the presence of multiple genes from the microcystin biosynthesis cluster, confirms the ongoing potential for toxin production in these systems. Sequencing results further support *Microcystis aeruginosa* as a key MC-producing taxon in the São Pedro reservoir, in agreement with previous findings from Portuguese freshwater environments.

Although measured MC concentrations were below established guideline values for drinking water, their recurrent occurrence highlights a chronic exposure scenario that may become more relevant under future conditions of increased temperature, drought frequency, and nutrient enrichment. Given the role of the Alqueva system for water supply, agriculture, and recreation, the implementation of regular monitoring programs that integrate chemical toxin analysis with the molecular detection of toxic cyanobacteria is essential. Such approaches will be critical for early warning, informed risk assessment, and the sustainable management of freshwater resources in a changing climate.

## 5. Materials and Methods

### 5.1. Selection of Reservoirs and Sampling Strategy

**Figure 1:**
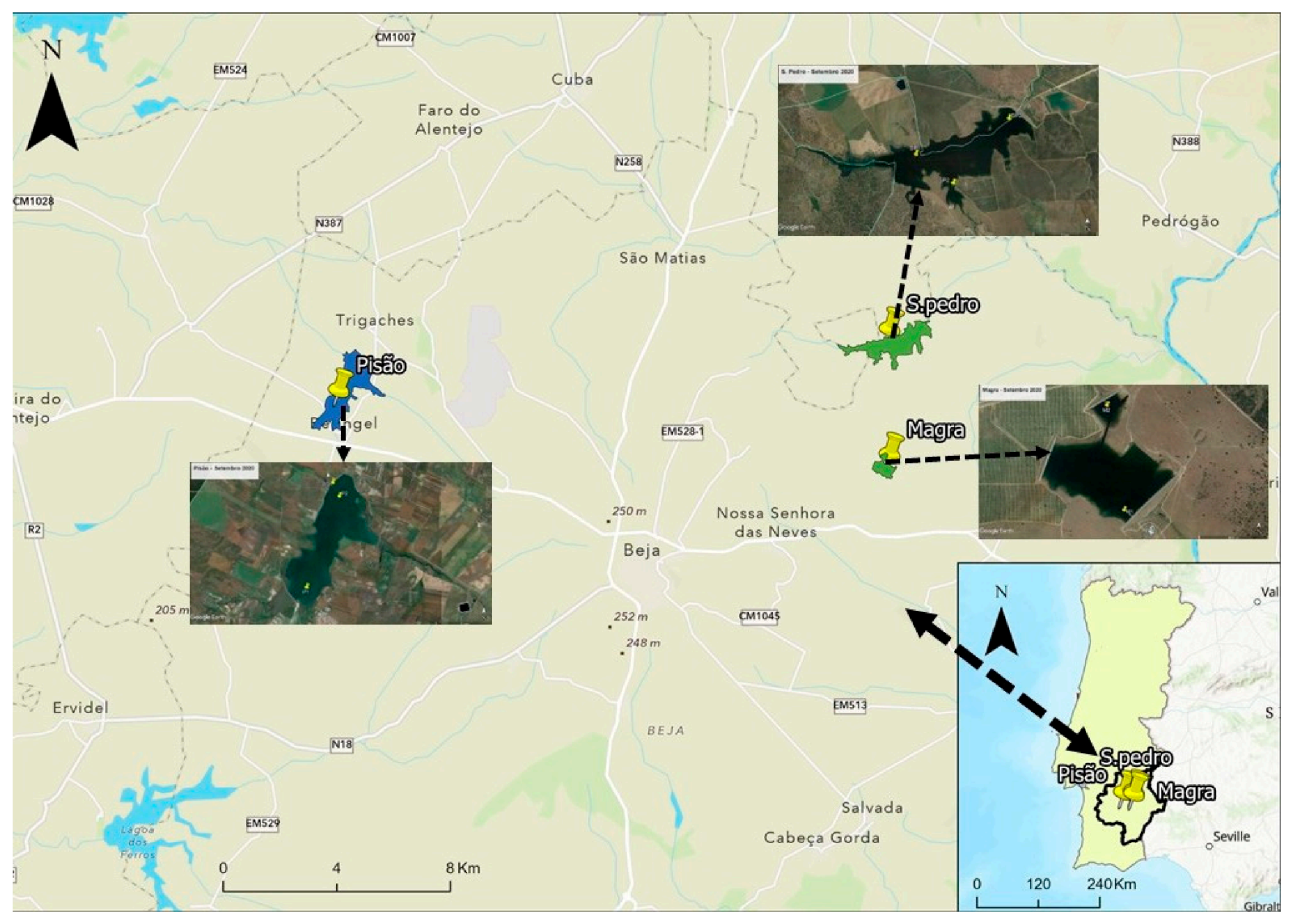
Location of the reservoirs—Pisão, S. Pedro and Magra—from the Alqueva multipurpose water system located in the Southern region of Portugal, monitored in this study. Basemap sources: Esri, Intermap, NASA, NGA, USGS, TomTom, Garmin, FAO, NOAA, © OpenStreetMap contributors, and the GIS User Community.

Three reservoirs from the Alqueva Water Storage System (Empreendimento de Fins Múltiplos de Alqueva, EFMA) were selected for this study. The selection was based on a preliminary risk assessment conducted using phytoplankton data kindly provided by the company EDIA (Beja, Portugal). All three reservoirs are located in the Beja region in southern Portugal and are integrated in the Pedrogão and Alqueva subsystems (Figure 1). For the study, two to three sampling points were chosen within each reservoir, as shown in Supplementary Figure S3. These points were located near the water inlet and outlet, and at the deepest part of the reservoirs. Sampling was conducted in 2020 and 2021 during the warmest months—July, August, and September—with one sampling event per month. This sampling period coincided with the time of highest cyanobacterial occurrence, according to the data provided by EDIA. Based on the reported cyanobacterial cell densities, the risk of toxic cyanobacteria occurrence in the São Pedro, Magra, and Pisão reservoirs was considered moderate to high.

### 5.2. Water Sampling

Sampling in the reservoirs was carried out with the aid of a boat, in the morning, between 7 am and 12 pm. At each sampling point, the physical-chemical parameters of the water, namely temperature, pH, salinity, and partial and absolute conductivity, were measured and recorded with a HANNA HI98194 multiparametric probe (Hanna Instruments, Villafranca Padovana, Italy). A turbidity test was carried out using a Secchi disk, at each sampling point to determine the depth of the euphotic zone. Secchi depth was measured using a Secchi disk, and the euphotic depth was estimated as 2.5 times the Secchi depth. The calculated euphotic depths ranged from 1 to 4 m. Three water subsamples were collected at different depths along the euphotic water column, using a Van Dorn hydrographic bottle. These were mixed (in a 16 L bucket) to make a composite sample from each sampling point. Water samples were stored in thermal boxes with ice to keep the samples refrigerated during transport to the laboratory, where these samples were stored.

### 5.3. Sample Treatment and Toxin Extraction

For chemical analysis, water samples (1 L) were filtered with a vacuum filtration system, using glass microfiber paper filters, 47 mm in diameter and 0.7 μm porosity (Fisherbrand, Loughborough, UK). The filters, which retained all suspended matter (including phytoplankton) as well as the filtered water were stored at −20 °C for further analysis. The extraction of toxins from the intracellular component of the biomass retained on the filters was performed as follows: the filters were ground in a mortar with liquid nitrogen and the powder was mixed with MeOH at 10% (*v*/*v*) (5 mL per filter) and sonicated using a Sonopuls HD-2070.2 sonicator equipped with a MS-72 probe (Bandelin, Berlin, Germany). Five sonication cycles of 1 min at 20 kHz and 25% amplitude were carried out in an ice bath. The homogenate was then centrifuged (13,000 g, for 20 min) and the supernatant recovered and dried in an acid-resistant CentriVap Concentration system (Labconco^®^, Kansas City, MO, USA) equipped with a Coldtrap at −50 °C, coupled to an RV5 Rotary Vane vacuum pump (Edwards, Crawley, UK).

The extraction of toxins from the filtered water was carried out with two different solid-phase extraction (SPE) methods. The samples from 2020 were processed as follows: Oasis^®^ HLB cartridges (3 cc/60 mg) were activated with 5 mL 100% MeOH and 5 mL ultrapure water, under vacuum using a 12-port SPE vacuum manifold. Filtered water samples were then loaded in the cartridges (0.5–1 L), and the toxins were eluted from the cartridges with 5 mL 95% MeOH and 5% (*v*/*v*) formic acid (FA). This method is suitable for the purification of the cyanotoxins microcystins. Water samples from 2021 were processed based on the method of Zervou et al. (2017) [35]. This method allows for the simultaneous purification and concentration of several types of cyanotoxins. Five mL of MeOH (100%) was added to 400 mL of sample and the pH was corrected to 11 with 2 M NaOH (*w*/*v*). Then a HyperSep PGC (porous graphitic carbon, 200 mg, 3 cc, 30–40 μm, Thermo Scientific, Loughborough, UK) was connected on top of an Oasis HLB (200 mg, 6 cc, 25–35 μm, Waters Corporation, Milford, MA, USA) and conditioned with 6 mL (10%, *v*/*v*) dichloromethane (DCM), followed by 6 mL MeOH (100%) and 6 mL water (pH11). Samples were loaded into the SPE assembly and the cartridges were dried for 15 min (air under vacuum). Afterwards, the position of the SPE cartridges was reversed and the analytes were eluted with 10 mL DCM:MeOH (40:60, *v*/*v*), containing 0.5% FA. The eluate was dried under a stream of nitrogen.

### 5.4. LC-MS Analysis

For LC-MS analysis, dried samples were resuspended in 1000 μL of 50% (*v*/*v*) MeOH and 0.1% (*v*/*v*) formic acid, using an ultrasound bath. Samples were next filtered with SFPE-22E-050 into 2 mL vials and analyzed using a Liquid Phase Chromatograph Alliance e2695 HPLC system, coupled to a triple quadrupole spectrometry detector (Micromass^®^ Quattro microTM API, Waters Corporation, Manchester, UK), with a electrospray ionization (ESI) source. The data acquisition and processing were carried out in the program MassLynx version 4.1. The mass spectrometer was operated in positive mode, and quantification done with multiple reaction monitoring (MRM). The conditions of analysis were the following: (a) Capillary voltage was 3.5 kV; (b) cone at 20 V; and (c) extractor at 3 V and lens at 0.2 V. The source temperature was 120 °C, desolvation 350 °C and 500 L/h. Nitrogen was used as a sheath and auxiliary gas and Argon as a collision gas at a pressure of 0.5 bar. Molecular separation was carried out on a C18 Hypersil Gold column (100 × 4.6 mm I.D., 5 μm, ThermoScientific, Waltham, MA, USA) kept at 45 °C, with a flow rate of 0.35 mL/min. The injected volume was 10 μL. MeOH was used as the mobile phase A and ultrapure water with 0.1% formic acid as the mobile phase B. Sample elution comprised the following steps: 5% A and 95% B for 3 min, increase to 40% A for 4 min and holding for 1 min, increase to 60% A for 7 min and holding for 2 min, increase to 80% A for 1 min and returning to initial conditions for 20 min. Analytical conditions were optimized using standard solutions and extracts of cyanotoxin-producing cyanobacteria. Transitions, cone, and collision energy voltages for each cyanotoxin are present in Supplementary Table S1. The target compounds were: Cylindrospermopsin (CYN), Anatoxin-a (ANTX), Saxitoxin (STX), Nodularin-R (NOD) and seven msicrocystin analogs (MC-RR, MC-YR, MC-LR, MC-LA, MC-LY, MC-LW and MC-LF). The toxin standards and samples were injected in duplicate and after each set of 10 samples a blank and two standards mix solutions of different concentration were run. Quantification was performed by external calibration curve. The MS operating parameters and mass transitions used for the identification of the different toxins are reported in Supplementary Table S1.

### 5.5. DNA Extraction and Molecular Analysis

For the molecular biology analysis, a variable volume of water was filtered to collect a sufficient amount of phytoplankton cells (between 1 and 3 L, depending on phytoplankton abundance). Genomic DNA (gDNA) was extracted using the PureLink™ Genomic DNA Mini Kit (Invitrogen, Waltham, MA, USA), following the manufacturer’s protocol for Gram-negative bacteria with minor modifications. For DNA elution, 50 μL of elution buffer was used. For PCR amplifications, the GoTaq^®^ G2 Flexi DNA Polymerase kit (Promega, Madison, WI, USA) was used. Each PCR reaction mixture comprised 4 μL of 5× Green GoTaq^®^ Flexi Buffer, 2 μL of 25 mM MgCl_2_, 2 μL of forward primer, 2 μL of reverse primer, 1 μL of 2.5 mM deoxynucleotide triphosphate mix (dNTPs), 0.5 μL of bovine serum albumin (BSA; 10 mg/mL), 0.1 μL of GoTaq^®^ DNA Polymerase (5 U/μL), and 1 μL of DNA template in a final reaction volume of 20 μL. The primer sets used for gene amplification are listed in Supplementary Tables S3 and S4. PCR reactions were performed in a Veriti™ 96-Well Thermal Cycler (Applied Biosystems, Foster City, CA, USA). Amplification products were confirmed by electrophoresis in 1.5% agarose gels. The PCR cycling conditions are detailed in Supplementary Table S5. Positive controls were included in all PCR assays, using DNA extracted from material provided by the LEGE CC culture collection (LEGE CC, https://lege.ciimar.up.pt, accessed on 23 October 2024). Genomic DNA from *Cylindrospermopsis raciborskii* LEGE 97047 was used to validate the presence of cyanobacterial DNA, and DNA from *Microcystis aeruginosa* LEGE 00063 was used to validate the occurrence of the genus *Microcystis* and genes belonging to the microcystin synthetase complex.

## Figures and Tables

**Table 1:** LC–MS screening results of the toxins MC-LR, MC-YR, MC-RR, CYN, STX, and ANTX in the suspended particulate fraction (intracellular toxins) of water samples collected from the Alqueva reservoirs São Pedro (SP), Magra (M), and Pisão (P). “B” refers to samples collected in 2020 and “A” to samples collected in 2021, “nd” means not detected.

	Sample	MC-LR	MC-YR	MC-RR	CYN	STX	ANA
July	SP	AB	A	AB	nd	nd	nd
	M	AB	A	B	nd	nd	nd
	P	B	AB	B	nd	nd	nd
August	SP	AB	nd	AB	nd	nd	nd
	M	AB	nd	B	nd	nd	nd
	P	B	B	B	nd	nd	nd
September	SP	B	nd	B	nd	nd	nd
	M	B	B	B	nd	nd	nd
	P	nd	B	B	nd	nd	nd

**Table 2:** Average concentrations of microcystins (µg/L) in the water samples collected in Alqueva reservoirs São Pedro (SP), Magra (M) and Pisão (P). (nd) not detected. (SD) Standard deviation.

		2020		2021					
	Sample	MC-LR	SD	MC-LR	SD	MC-YR	SD	MC-RR	SD
July	SP	0.062	0.033	0.062	0.006	nd	nd	0.044	0.011
	M	0.036	0.002	0.067	0.004	nd	nd	0.044	0.005
	P	0.019	0.002	0.031	0.002	0.013	0.018	0.004	0.006
August	SP	0.060	0.006	0.053	0.002	nd	nd	0.031	0.001
	M	0.053	0.001	0.056	0.001	nd	nd	0.036	0.008
	P	0.017	0.002	0.032	0.002	0.013	0.019	0.013	0.000
September	SP	0.115	0.131	0.036	0.002	nd	nd	0.014	0.000
	M	0.023	0.004	0.044	0.001	0.020	0.020	0.021	0.001
	P	0.019	0.005	0.000	0.000	0.012	0.018	0.013	0.000

**Table 3:** Detection of 16S rRNA gene sequences specific to *Microcystis* sp. by PCR, using the pair of primers Micr 184F/Micr 431R. (+) indicate samples where genes were detected. In contrast, absence of symbol indicates samples where genes were not detected.

Month/Location	S. Pedro			Magra		Pisão	
	SP1	SP2	SP3	M1	M2	P2	P3
July	+	+	+	+			
August	+	+	+		+		+
September	+	+	+	+	+	+	+

**Table 4:** The detection of gene sequences from the microcystin biosynthesis gene cluster (*mcyABCDEG*) by PCR. (+) indicates samples where genes were detected. In contrast, the absence of symbol indicates samples where the genes were not detected.

			General		*Microcystis* sp.			
		Sample	*mcyA*	*mcyB*	*mcyB*	*mcyD*	*mcyE*	*mcyG*
S.Pedro	SP1	J	+	+		+	+	+
		A	+	+		+		+
		S		+				
	SP2	J		+		+	+	+
		A	+	+	+	+	+	+
		S	+	+		+	+	
	SP3	J	+	+		+	+	+
		A	+	+		+		+
		S	+	+			+	
Magra	M1	J	+	+	+			+
		A						
		S	+	+	+	+	+	
	M2	J						
		A	+	+		+	+	+
		S	+	+	+	+		
Pisão	P2	J						
		A						
		S	+	+			+	
	P3	J						
		A			+		+	+
		S	+	+	+		+	+

**Table 5:** BLAST search results, of the gene sequence amplified by the pair of primers of the gene *mcyA* (CD1F/CD1R), in S. Pedro reservoir (SP).

Month	Sample	e-Value	% Identity	Identification	Accession Number
July	SP1	8.00 × 10^−111^	100	*Microcystis* *aeruginosa* NIBR18	CP086723.1
	SP2	8.00 × 10^−111^	99.56		
August	SP1	5.00 × 10^−103^	100		
	SP2	8.00 × 10^−106^	100		
**Sequenced gene fragment:** *mcyA* (general)					
CD1F	AAAATTAAAAGCCGTATCAAA				
CD1R	AAAAGTGTTTTATTAGCGGCTCAT				

## Data Availability

The original contributions presented in this study are included in the article/Supplementary Material. Further inquiries can be directed to the corresponding author.

## Supplementary Materials

The following supporting information can be downloaded at: https://www.mdpi.com/article/10.3390/toxins18100430/s1, Figure S1: MRM chromatograms and respective transitions of MC-LR in the SP3 sample from July. (A) Extracted ion chromatogram (XIC) of the 995.5 > 599 transition; (B) Extracted ion chromatogram (XIC) of the 995.5 > 135 transition; (C) MRM total ion chromatogram (TIC), with the peak area associated with MC-LR highlighted in blue for quantification.; Figure S2: Results of agarose gel electrophoresis of amplified DNA fragments corresponding to *mcyD* (*Microcystis* sp.-specific). On the left, the molecular weight marker, followed by PCR amplification products from the environmental samples identified at the top. C+—positive control; C−—negative control. Samples showing a luminescent band with the same molecular weight as the C+ are considered positive, whereas the remaining samples are negative for the fragment associated with the *mcyD* gene; Figure S3: Sampling points in S. Pedro (a), Magra (b) and Pisão (c) reservoirs and respective GPS coordinates; Table S1: Detection of cyanobacteria 16S rRNA and Phycocyanin gene sequences by PCR, using respectively the pair of primers 27F/209R and PCβF/PCβR. (+) indicate samples where both genes were detected. In contrast, absence of symbol indicate samples where these genes were not detected; Table S2. Mass-spectrometry operating parameters for detection and quantification of cyanotoxins; Table S3. Primer sets used in PCR for the detection of cyanobacteria (16S rRNA and the phycocyanin operon), and of the 16S rRNA gene of Microcystis [18,19,36]; Table S4. Primer sets used in PCR for the detection of genes responsible for MC synthesis, without taxonomic distinction (general), and in *Microcystis* sp. [20,21,37,38,39]; Table S5. PCR program settings applied for the detection of cyanobacteria, *Microcystis* sp., and microcystin-producing genes.
