# Supplementary material for: Occurrence of Microcystins and Toxin-Producing Cyanobacteria in Reservoirs of the Alqueva Water Storage System (Southern Portugal)
Source: Toxins (Basel). 2026 Oct 7;18(10):430. doi: 10.3390/toxins18100430 (PMC13709596; doi:10.3390/toxins18100430)
Supplement: Supplementary file 1 [file toxins-18-00430-s001.zip › toxins-4529627-supplementary.pdf]

Supplementary Materials: Occurrence of Microcystins and Toxin-Producing Cyanobacteria in Reservoirs of the Alqueva Water Storage System (Southern Portugal)

Tomé Azevedo, Lucas Ribeiro, Joana Azevedo, Rui Barbosa, Rita Mendes, Joana Martins, Marisa Freitas, Vitor Vasconcelos and Alexandre Campos

Results:

Table S1. Detection of cyanobacteria 16S rRNA and Phycocyanin gene sequences by PCR, using respectively the pair of primers 27F/209R and PCβF/PCβR. (+) indicate samples where both genes were detected. In contrast, absence of symbol indicate samples where these genes were not detected.

Month/Location	S. Pedro				Magra		Pisão			
	**SP1**	**SP2**	**SP3**		**M1**	**M2**	**P1**	**P2**	**P3**	**P4**
July	+	+	+		+					
August	+	+	+			+			+	
September	+	+	+		+	+		+	+	

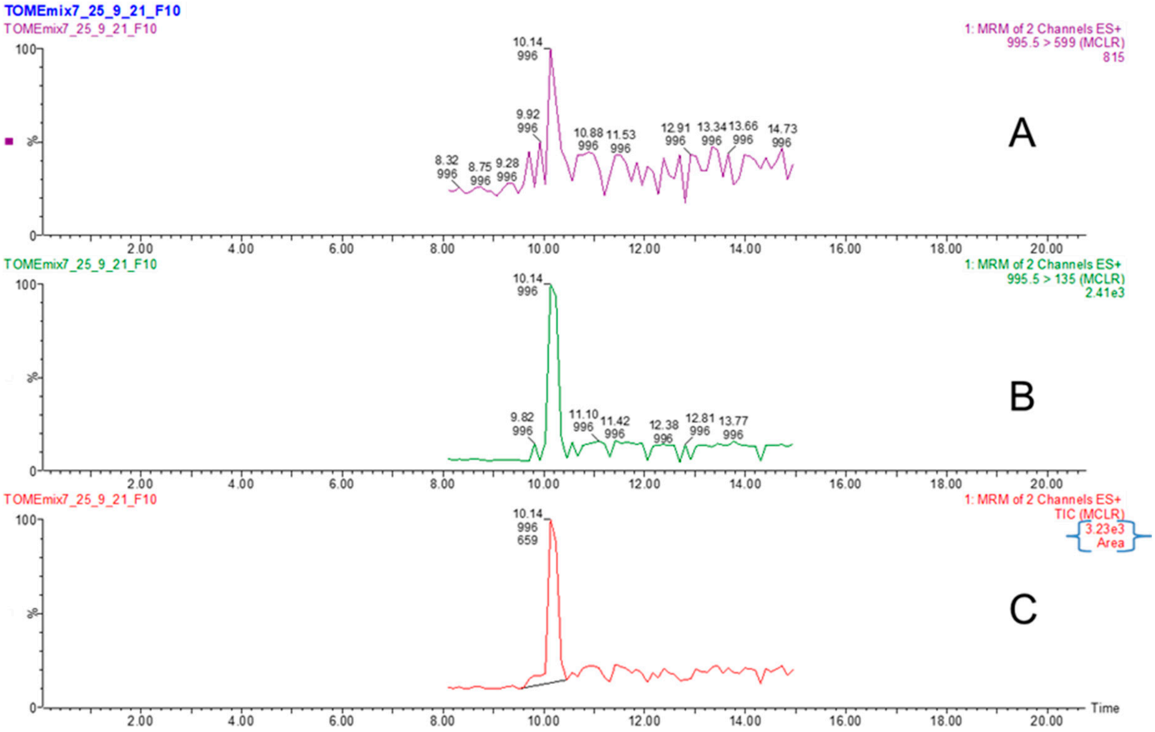

Figure S1. MRM chromatograms and respective transitions of MC-LR in the SP3 sample from July. A) Extracted ion chromatogram (XIC) of the 995.5 > 599 transition; B) Extracted ion chromatogram (XIC) of the 995.5 > 135 transition; C) MRM total ion chromatogram (TIC), with the peak area associated with MC-LR highlighted in blue for quantification.

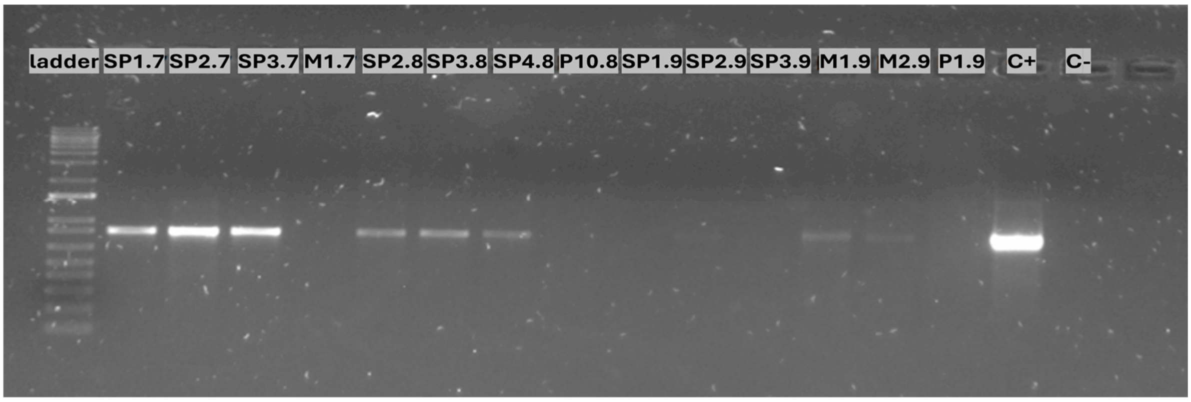

Figure S2. Results of agarose gel electrophoresis of amplified DNA fragments corresponding to mcyD (Microcystis sp.-specific). On the left, the molecular weight marker, followed by PCR amplification products from the environmental samples identified at the top. C+ – positive control; C− – negative control. Samples showing a luminescent band with the same molecular weight as the C+ are considered positive, whereas the remaining samples are negative for the fragment associated with the mcyD gene.

Methods:

a)

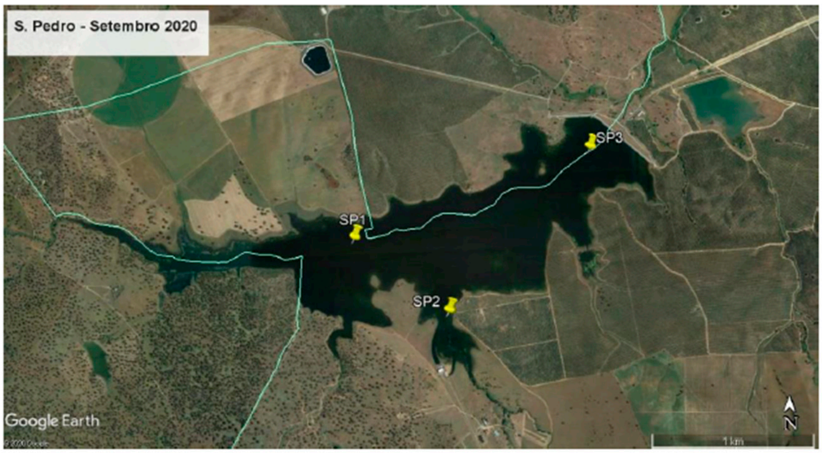

SP1 – 38°05'04.7"N 7°46'08.5"W; SP2 – 38°05'11.4"N 7°45'36.7"W; SP3 - 38°05'23.7"N 7°45'10.1"W

b)

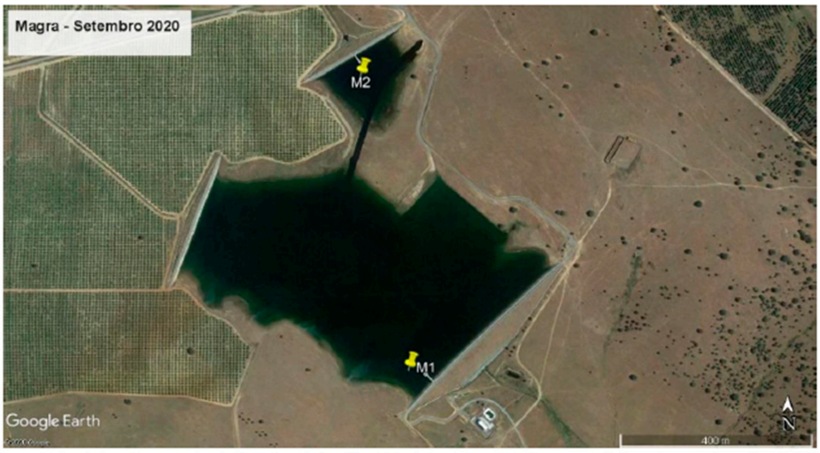

M1 – 38°02'58.0"N 7°46'04.8"W; M2 – 38°02'43.4"N 7°46'00.8"W

c)

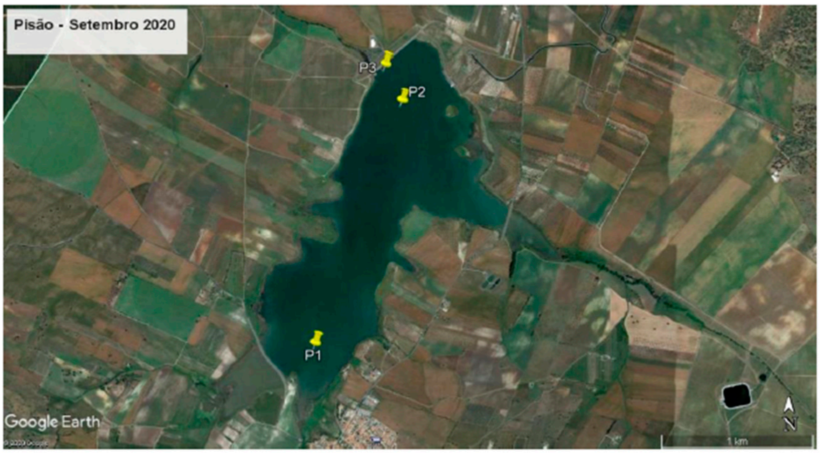

P1 - 38°04'43.0''N, 7°58'50.6''W; P2 -38°04'21.7''N, 7°58'29.8''W; P3 - 38°03'48.6''N, 7°59'15.9''W

Figure S3. Sampling points in S. Pedro (a), Magra (b) and Pisão (c) reservoirs and respective GPS coordinates. .

Table S2. Mass-spectrometry operating parameters for detection and quantification of cyanotoxins.

Cyanotoxin	Mass-charge transition (*m/z*)	Colision energy	Limit of detection (µg/L)
Anatoxin-a	166>131<br>166>149	20<br>25	5,7 ± 0,3
Cylindrospermopsin	416>336<br>416>194	20<br>28	3,8 ± 0,7
Saxitoxin	300>282<br>300>204	20<br>25	7,9 ± 1,3
Microcystin-LR	995.5>135<br>995.5>599	50<br>50	2,9 ± 0,8
Microcystin-RR	520>70<br>520>135	50<br>50	4,1 ± 0,3
Microcystin-YR	1045.5>135<br>1045.5>175	50<br>50	3,2 ± 0,8

Cyanotoxin	Retentio Time	Mass-to-charge ratio (*m/z*) transition	Cone (V)	Collision energy (V)
Microcystin-YR	12.90	1045.5>13.2<br>1045.5>599	20<br>20	60<br>55
Microcystin-Lw	16.22	1047.3>579.5	50	20
Microcystin-La	15.49	932.5>135<br>932.5>599.1	50<br>50	20<br>20
Microcystin-LR	13.25<br>15.00<br>(gradient 1 and2)	996.5>135.05<br>995.5>599.2	50<br>50	30<br>30
Microcystin-RR	12.30	520.3>105.1<br>520.3>135.1	20<br>20	40<br>30
Microcystin-LF	16.16	1008.6>515.1	50	20
Microcystin-Ly	15054	1024.5>677.2	50	20
Nodularin-R	12.98	825.3>135.5	50	20
Cilindrospermopsin	3.9016	415.9>176	20	80

Table S3. Primer sets used in PCR for the detection of cyanobacteria (16S rRNA and the phycocyanin operon), and of the 16S rRNA gene of *Microcystis.*

Gene	Primer	Primer sequence 5´-3´	Sequence size (base pairs)	Reference
16S rRNA cyanobateria	27F	AGAGTTTGATCCTGGCTCAG	780	[18], [36]
	809R	GCTTCGGCACGGCTCGGGTCGATA		
	740F	GGCYRWAWCTGACACTSAGGGA	754	[18]
	1494R	TACGGTTACCTTGTTACGAC		
Operon phycocyanin	PC?F	GGCTGCTTGTTTACGCGACA	700	[19]
	PC?R	CCAGTACCACCAGCAACTAA		
16S Rrna Microcystis sp.	Mic 184F	GCCGCRAGGTGAAAMCTAA	220	[18]
	Mic 431R	AATCCAAARACCTTCCTCCC		

Table S4. Primer sets used in PCR for the detection of genes responsible for MC synthesis, without taxonomic distinction (general), and in *Microcystis* sp.

Gene		Primer	Primer sequence 5´-3´	Sequence size (base pairs)	Reference
Geral	mcyA	CD1 F	AAAATTAAAAGCCGTATCAAA	297	[37]
		CD1 R	AAAAGTGTTTTATTAGCGGCTCAT		
	mcyB	FAA	CTATGTTATTTATACATCAGG	758	[38]
		RAA	CTCAGCTTAACTTGATTATC		
Microcystin sp.	mcyA	MSF	ATCACGCAGTTGAGCAAGC	1300	[39]
		MSR	TGCAGATAACTCCGCA		
	mcyB	2156-F	ATCACTTCAATCTAACGACT	955	[21]
		3111-R	AGTTGCTGCTGTAAGAAA		
	mcyC	PSCF1	GCAACATCCCAAGAGCAAAG	674	[20]
		PSCR1	CCGACAACATCACAAAGGC		
	mcyD	PKDF1	GACGCTCAAATGATGAAAC	647	[20]
		PKDR1	GCAACCGATAAAAACTCCC		
	mcyE	PKEF1	CGCAAACCCGATTTACAG	755	[20]
		PKER1	CCCCTACCATCTTCATCTTC		
	mcyG	PKGF1	ACTCTCAAGTTATCCTCCCTC	425	[20]
		PKGR1	AATCGCTAAAACGCCACC		

Table S5. PCR program settings applied for the detection of cyanobacteria, *Microcystis* sp., and microcystin-producing genes.

Gene-alvo		Initial denaturing		35 cycles Denaturing		35 cycles Annealing		35 cycles Extension		Final extension	
		Temp.	Time	Temp.	Time	Temp.	Time	Temp.	Time	Temp.	Time
16S rRNA Cyanobacteria		92 °C	2 min	92 °C	20 seg	50 °C	30 seg	72 °C	1 min	72 °C	5 min
Operon phycocyanin		94 °C	3 min	94 °C	30 seg	50 °C	30 seg	72 °C	30 seg	72 °C	5 min
16S rRNA Microcystis sp.		95 °C	2 min	95 °C	90 seg	56 °C	30 seg	72 °C	50 seg	72 °C	7 min
Microcystin SP.	mcy A	95 °C	2 min	95 °C	90 seg	56 °C	30 seg	72 °C	50 seg	72 °C	7 min
	mcy B	94 °C	2 min	93 °C	10 seg	50 °C	20 seg	72 °C	1 min	72 °C	5 min
	mcy A	95 °C	2 min	95 °C	90 seg	60 °C	30 seg	72 °C	50 seg	72 °C	7 min
	mcy B	94 °C	5 min	95 °C	1 min	52 °C	30 seg	72 °C	1 min	72 °C	7 min
	mcy C	94 °C	5 min	95 °C	1 min	52 °C	30 seg	72 °C	1 min	72 °C	7 min
	mcy D	94 °C	5 min	95 °C	1 min	52 °C	30 seg	72 °C	1 min	72 °C	7 min
	mcy E	94 °C	5 min	95 °C	1 min	52 °C	30 seg	72 °C	1 min	72 °C	7 min
	mcy G	94 °C	5 min	95 °C	1 min	52 °C	30 seg	72 °C	1 min	72 °C	7 min
